# Milk and Protein Intake by Pregnant Women Affects Growth of Foetus

**DOI:** 10.3329/jhpn.v31i4.19991

**Published:** 2013-12

**Authors:** Fatemeh Borazjani, Kambiz Ahmadi Angali, Shanuak S. Kulkarni

**Affiliations:** ^1^School of Health, Bushehr University of Medical Sciences, Iran; ^2^Department of Biostatistics, Schools of Health, Ahvaz Jundishapur University of Medical Sciences, Iran; ^3^Department of Anthropology, University of Pune, India

**Keywords:** Anthropometric measurements, Foetal growth pattern, Growth velocity of foetus, Maternal daily protein intake, Maternal milk intake, India

## Abstract

The study assessed the effects of the daily intake of milk and protein by pregnant women on foetal growth and determined the growth pattern and velocity of growth. A total of 504 ultrasound observations from 156 respondents were collected following a cross-sectional design in the last trimester of pregnancy; majority of them were in the last month of pregnancy. *De facto* and purposive sampling was done, and direct interviews of affluent pregnant women were conducted. Kruskal-Wallis test shows that majority of the respondents had tendency to consume 155.65 to 465.17 mL of milk per day, resulting in better and higher foetal growth. Most respondents consumed about 50-70 g of protein per day, and the foetal growth measurements, such as abdomen-circumference, femur length, biparietal diameter, and head-circumference, on an average, were higher in the same group. Quadratic regression model exhibited that all the traits of growth pattern in Model 1 (low milk and protein intake) appeared to have more mode of decline, in contrast to Model 2 (more milk and protein intake), which shows better growth. In addition, velocity of growth pattern was obtained through the first derivative of quadratic regression of growth pattern. Moreover, 95% confidence interval calculated for regression line slope of Model 1 and Model 2 showed that the estimation point (2 B_2_) of Model 1 does not lay into 95% CI of Model 2; so, statistical significance assorted and also the same trend conversely hold for Model 2. The rate of growth was highly influenced by maternal milk and protein intake. These findings suggest that contribution of common nutrients or other nutritional factors present in milk and protein promote the growth of foetus.

## INTRODUCTION

A developing foetus needs protein to build the cells of its body. The maternal diet supplies all the proteins that a baby needs; so, if the diet of a pregnant woman is deficient, her baby can suffer. The baby grows more rapidly during the second and third trimester; hence, the protein levels during the latter half of the pregnancy is more important than earlier in the development of foetus. Epidemiological (1-3) and experimental (4-7) studies show that the increased consumption of protein during pregnancy resulted in foetal growth retardation. In rodent and sheep, higher pre-gestational protein consumption leads to impaired foetal growth as well (8-10).

A restricted-protein diet during conception also causes growth retardation; it is associated with reduced nutrient supply to the foetus (2,11). Findings of rodent studies also show that low protein intake during gestation can result in low birthweight and subsequently leads to various metabolic disturbances (12). Milk is also an efficient food for the delivery of many nutrients (proteins, minerals, vitamins, or combination of these) essential for foetal development and, therefore, of potential importance for linear foetal growth. Consumption of milk also increases blood concentration of insulin, like growth factor I (IGF I) which is a major determinant of growth (13). There are a number of studies, including prospective and retrospective observational and randomized intervention trials which revealed that there is a positive association between intake of dairy products, especially milk, with birth parameters of the offspring. Amongst a mass of evidence in literature, only Chang *et al.* (14) established the role of milk on growth of femur length of foetus, showing that higher milk intake is related to better growth of femur length of foetus. This cross-sectional study is performed to assess the role of maternal milk and protein intake with its lower and upper limit during conception on growth of foetus and to highlight the potential role and net effect of milk and protein with reference to limited and excess amounts on growth of foetus during gestational period.

## MATERIALS AND METHODS

This cross-sectional study was carried out in Pune, Maharashtra state, India. Pregnant mothers (n=156) with 504 ultrasound measurements of the abdomen-circumference (AC), head-circumference (HC), biparietal diameter (BPD), and femur length (FL) from 16 to 38 weeks of gestation were identified through antenatal clinics of two hospitals during 2009. The respondents were approached in their last trimester of pregnancy, and majority of them were in the last month of pregnancy, which was close to time of delivery. To prevent dropout of subjects, normal mothers with healthy foetus were selected. In addition, baby's birth-record consisted of baby's head-circumference, birth-length, birthweight and gender of the newborns available in the obstetric department of related hospital. The maternal sonography reports were also available in their documents, to make better possibility to recruit the subjects. Moreover, mothers were not aware about the gender of foetus during gestation. It was not mentioned in sonography reports. By knowing the gender of the offspring, according to the hospital birth-records, there was a possibility of (biased) gender differences in foetal parameters and maternal diet analysis.

All participants in the study were drawn from among affluent women who were being referred to the obstetric department of hospitals for monthly check-up. This group of pregnant women was from better socioeducational level and paying more attention to their situation during entire course of pregnancy. Affluent mothers covered all the criteria of sample selection. To participate in the study, women had to meet the following inclusion criteria: (i) at least three sonography reports since second trimester to third trimester; (ii) ultrasound measurements of HC, BPD, AC, and FL; and (iii) singleton pregnancy. Exclusion criteria of sampling included: (i) any family history of congenital disease, (ii) suffering from chronic disease, and (iii) any complicated position of foetus during pregnancy. Written informed consent forms were collected from all the subjects, and the study was approved by medical manager of hospitals and ethical committee of the University of Pune. The research questionnaire was constructed with three sections: (i) demographic background, (ii) maternal gestational status and medical history of mother, and (iii) dietary data.

The questionnaire was pretested in a pilot study among 50 pregnant mothers from the sample based on milk consumption; the total sample-size calculated was 156. Cronbach's alpha used in evaluating the validity of the questionnaire (Cronbach's alpha=0.76) indicated adequate consistency. Milk was considered rich in protein and nutrients due to the presence of vitamins, trace elements, and hormones, which are the most important factors in the promotion of growth. The demographic backgrounds of mothers and their spouse, including information on religion, caste, place of residency, maternal age, family type, educational status, income level, and occupation, were recorded. In addition, gestational weight gain, parity, and self-reported pre-pregnancy weight and height were also recorded. Body mass index was calculated (weight in kg/height in metre squared).

### Dietary assessment

Maternal dietary evaluation was carried out by a 24-hour recall method and food frequency questionnaire (FFQ). Respondents were asked to recall all main daily foods, snacks, and beverages that were consumed during the past 24 hours. The food frequency questionnaire, based on the pattern of Indian foodstuffs, was formulated. The main food items were divided into eight categories (cereal; pulses and legume; milk products; leafy vegetables; other vegetables; fruits; meat products, including egg, fish, dry fish, chicken, mutton; and oils. Additional items were also incorporated according to maternal consumption. Each category of foodstuff was attributed to frequency of intake varying from daily, weekly, monthly, rarely, never and also to the different portion-sizes (small, medium, and large). In these approaches, women were asked for the usual frequency in which specific foods were consumed over time. The macronutrient contents of foods were extracted from nutrient value of Indian foods (15).

### Statistical analysis

**Figure 1.**
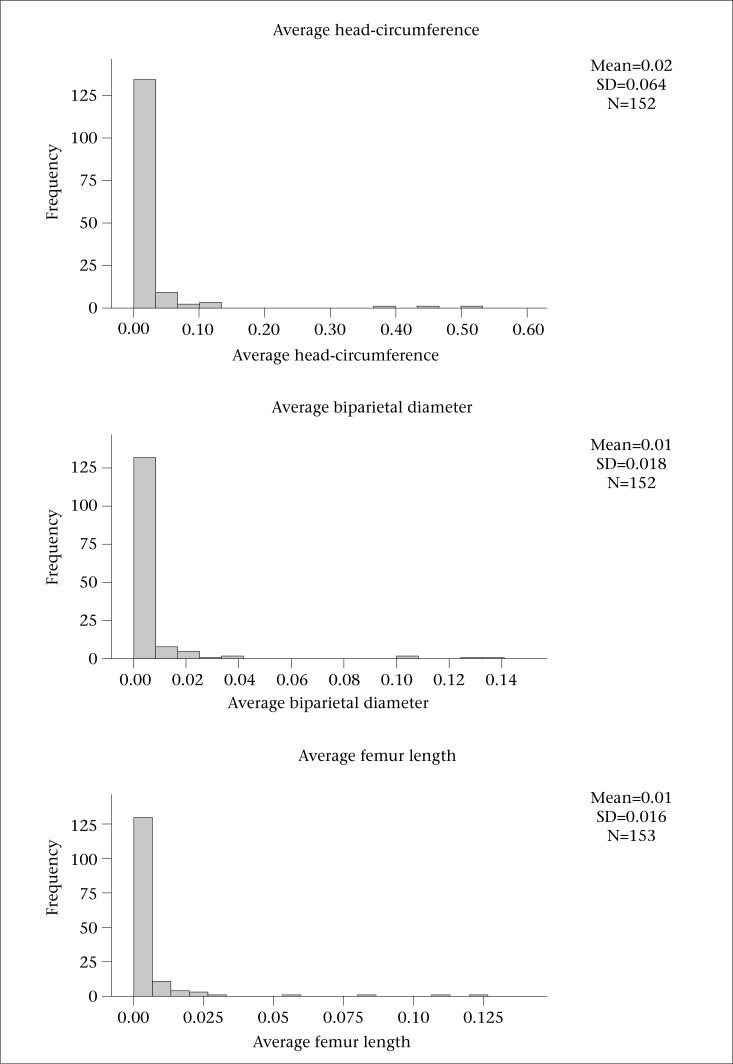
Histogram of foetal growth parameters

The information was compiled using SPSS (version 16). Statistical analyses comprising descriptive and inferential statistics were carried out. At commencement, descriptive analysis was done to express mean, standard deviation, standard error of mean, and the minimum and maximum of continuous variables that consisted of: maternal intake of macronutrients and micronutrients and percentage of energy derived from macronutrients intake. The non-parametric Kruskal-Wallis test was carried out to illustrate the effects of milk intake on growth parameters of foetus. Application of this test was based on the related histograms of growth measurements of foetus (Figure 1). It indicates that there is no normal distribution through ANOVA test for each variable, including foetal parameters (average AC, average BPD, average FL, and average HC). The milk intake was categorized based on average intake and standard deviation (SD) as follows:

Group 1: ≤Mean–SD [≤155.64 mL/day]
Group 2: Mean–SD to Mean+SD [155.65 to 465.17 mL/day]
Group 3: ≥Mean+SD [≥465.18 mL/day]

For all measurements, regression analysis was applied to examine linear and quadratic models to identify relationship between anthropometric parameters of foetus with gestational age. The model chosen was the one that gives the best fit to the data, in which the models were chosen based on the coefficient of determination R^2^.

The general form of model is given as y_t_=b_0_+b_1_t+b_2_t^2^ where t is gestational age 14^th^-40^th^, and b_0_, b_1_, b_2_ are regression coefficients and are estimated through least-square method. The y_t_ denotes the corresponding biometric measurements (BM), including BPD, HC, AC, and FL at age t. Based on regression analysis, y_t_ at age t (y_t_|t) has normal distribution, with mean b_0_+b_1_t+b_2_t^2^ and variance MSE (mean square error). Polynomial regression coefficients, MSE, and R^2^ for each model have been given separately (Table 1). The R^2^ is considered an appropriate model-indicator; therefore, a high value of R^2^ is desirable and leads to the variability of each BM estimated through regression model properly. Velocity model is another statistical model to evaluate velocity of growth, constructed by taking derivative of polynomial regression model (dy_t_/dt) where ‘d’ is a mathematical abbreviation that stands for derivative of foetal measurements in respect of the gestational age and, therefore, v_t_=b_1_+2b_2_t where v_t_ is the velocity of BM at age t, b_1_ and b_2_ are the same as values obtained by polynomial regression model, and 2b_2_ is the velocity rate of BM as t increases per unit.

**Figure 2.**
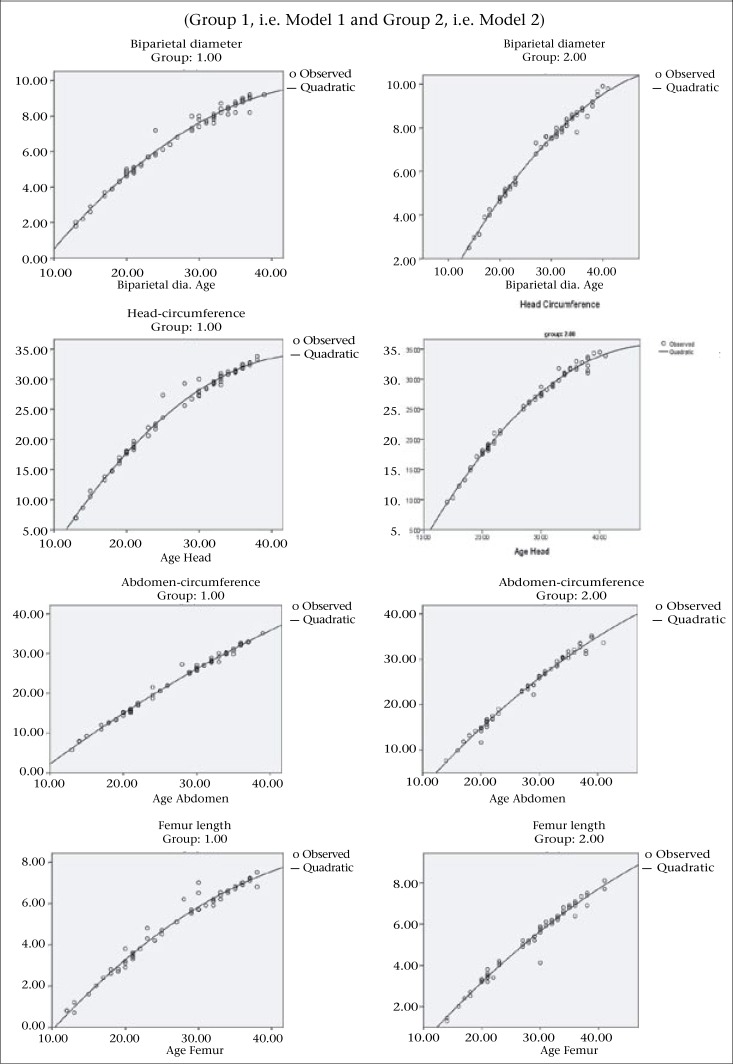
Foetal biometrical parameters

Dietary data were analyzed in two models. Model 1 is related to pregnant mothers who consumed milk and protein at low level (≤155.64 mL milk and ≤50 g protein/day) while Model 2 is associated with high level (≥465.18 mL milk and ≥70 g protein/day). Subsequently, to better illustrate the association between maternal daily nutrient intake and growth of biometrical parameters, we developed the test based on 95% confidence interval for Model 1 and 2. Also, a separate graph of each model for each foetal parameter is drawn in order to exhibit the variation of foetal parameters in gestational age of sonography observations on respondents in the same trimester of pregnancy (Figure 2).

## RESULTS

The mean age of the respondents was 28 years. Majority of women were Hindu (87.7%), and the rest were Christian (6.2%) and Muslim (5.2%); 90.6% of the respondents were in the high-income category, and the rest were in low-income category. More than half of the respondents, i.e. 51.3% had normal pre-pregnancy BMI. Also, majority of them were primiparae.

### Maternal dietary intake

The normal protein requirements during pregnancy as per ICMR (16) is 65 g/day. The study subjects consumed 63.33 g protein/day on an average, which is close to the recommended dietary allowance (RDA). As per ICMR, for an Indian pregnant woman doing sedentary work, the daily energy need is 300 kcal/day more than that for non-pregnant adult women, i.e. 1,875+300=2,175 kcal. So, the mean maternal energy intake (2084.80 kcal/day) is less than RDA but, with a slight difference. Normally, people should get the 50-60% of energy from carbohydrate; and 15-20%, 25-30% from protein and fat respectively (Table 2).

### Effect of maternal milk intake on growth of foetus

Maternal milk consumption is classified according to average milk intake and the standard deviation. The result through Kruskal-Wallis test shows that the majority of respondents had tendency to consume 155.65 to 465.17 mL of milk per day; better and higher foetal growth measurements were seen in that group (Table 3). Thereby, the Kruskal-Wallis test and regression mode 1 (reported elsewhere) fitted in maternal milk intake versus growth parameters in the newborn and foetus show the positive contribution of maternal milk intake in increasing trend in birthweight and foetal growth parameters (AC, BPD, HC, and FL). Enhancing the maternal milk intake during pregnancy results in a rise in birthweight and foetal growth parameters. This effect may be related to macronutrients, micronutrients, and minerals available in milk.

**Table 1.** Quadratic regression coefficient, R^2^, and MSE of two models

Parameter	Model 1					Model 2				
	B_0_(SE)	B_1_(SE)	B_2_(SE)	R^2^	MSE	B_0_(SE)	B_1_(SE)	B_2_(SE)	R_2_	MSE
HC	-18.84 (1.31)	2.34 (0.105)	-0.026 (.002)	0.99	0.532	-15.45 (1.154)	2.068 (0.087)	-0.021 (.002)	0.993	0.337
AC	-12.113 (1.15)	1.507 (0.091)	-0.008 (0.002)	0.994	0.385	-13.13 (1.738)	1.6 (0.135)	-0.01 (0.002)	0.987	0.071
FL	-4.4.1 (0.393)	0.468 (0.032)	-0.004 (0.001)	0.982	0.064	-3.027 (0.487)	0.352 (0.037)	-0.002 (0.0006)	0.979	0.063
BPD	-4.881 (0.434)	0.602 (0.035)	-0.006 (0.001)	0.986	0.06	-3.611 (0.408)	0.503 (0.031)	-0.004 (0.001)	0.989	0.042

### Impact of maternal protein intake on growth of foetus

To identify the effect of protein intake on growth of foetus, the maternal protein consumption was categorized into three classes: <50 g/day, 50-70 g/day, and >70 g/day. The descriptive analysis shows that half of the respondents were in the second group, and the other half in the first and the third group. Accordingly, Kruskal-Wallis test was carried out as an appropriate approach to observing how the foetal measurements would be influenced by maternal protein intake during pregnancy. Table 4 shows that the majority of respondents consumed about 50-70 g protein per day, and the foetal growth measurements, such as abdomen-circumference, femur length, biparietal diameter, and head-circumference, on average, were higher in the same group compared to the first and the third group. In addition, there was a strong positive significant contribution of maternal protein intake during pregnancy towards all the four foetal parameters measured in this study. Moreover, result shows a direct significant association between protein and energy intakes during pregnancy. Most respondents consumed animal protein less frequently. Therefore, consuming milk among vegetarian and non-vegetarian was treated as intake of protein-rich food.

### Growth pattern and velocity

Raw data were fitted adequately by quadratic polynomial regression in two models for all biometric measurements. R^2^ values for all regressions were higher than 0.97, indicating high quality of regression model. Table 1 and 5 exhibit the estimated values and standard errors of polynomial regression coefficients for all biometric measurements in two models.

Quadratic regression of foetal growth parameters with no sex-specific difference indicates that the function of the total daily protein and milk consumption during gestational period has a positive effect on foetal growth measurements. Hence, the distribution of subjects into two levels of dietary intake (Model 1 indicating lower intake, Model 2 indicating higher intake) proceeded to establish a growth difference under two levels of maternal dietary intake, in which foetal measurements were considered dependent variables, and gestational age was taken as the independent variable. Subsequently, growth pattern for all traits in Model 1 appeared to have more mode of decline in contrast to Model 2, which reflects better tendency of growth. Identically, the rate of growth was highly influenced by maternal dietary intake. Provided that the difference for a given model on foetal parameter, except for AC, was not significantly affected by these two models, a 95% confidence interval was calculated for regression line slope of Model 1 and Model 2 to approve this result. Result shows that the estimation point (2 B_2_) of Model 1 does not lay into 95% CI of Model 2; so, statistical significance assorted and also the same trend conversely hold true for Model 2. According to this rule, there is no significant difference in abdomen-circumference among these two models. Corresponding graphs associated with the models are given in Figure 2 and Table 1, which explain the rate of growth among two levels of maternal food intake.

**Table 2.** Maternal macronutrients intake

Descriptive statistics	% Energy derived from carbohydrate	Carbohydrate (g/d)	% Energy derived from protein	Protein (g/d)	% Energy derived from fat	Fat (g/d)	Energy (kcal/d)
Mean	62.68	328.95	12.74	63.33	25.02	57.91	2,084
Standard deviation	5.45	75.44	4.85	14.68	4.78	15.81	416.01
Variance	29.72	5,690.80	23.49	215.67	22.89	250	1,73,066.5
Minimum	35.21	154.43	8.78	33.10	13.49	25.2	1205
Maximum	76.10	553.03	68	105	50.31	111.90	3,380

**Table 3.** Association between maternal milk intake with foetal growth parameters

Foetal measurements against protein intake category	N	Mean foetal measurements (mm)	Standard deviation	p value
Average head-circumference for taking				
<155.64 mL	33	0.0166	0.0219	
155.65 to 465.17 mL	100	0.0260	0.0779	0.004
>465.17 mL	19	0.0178	0.0108	
Total	152	0.0229	0.0641	
Average abdomen-circumference for taking				
<155.64 mL	33	0.0192	0.0259	
155.65 to 465.17 mL	100	0.0289	0.0782	0.010
>465.17 mL	19	0.0209	0.0121	
Total	152	0.0259	0.0652	
Average femur length for taking				
<155.64 mL	33	0.0043	0.0055	
155.65 to 465.17 mL	100	0.0069	0.0191	0.006
>465.17 mL	19	0.0050	0.0030	
Total	152	0.0061	0.0158	
Average biparietal diameter for taking				
<155.64 mL	33	0.0049	0.0062	
155.65 to 465.17 mL	100	0.0076	0.0218	0.014
>465.17 mL	19	0.0049	0.0028	
Total	152	0.0067	0.0179	

## DISCUSSION

The major findings of the study through different analysis (Kruskal-Wallis and quadratic regression) revealed that growth parameters of foetus are affected by milk and daily total protein intake by mothers. Protein and milk consumption was categorized into three levels. Most of the subjects belonged to the second category of the intake of milk (i.e. 155.65 to 465.17 mL) and protein (50-70 g/day).

**Table 4.** Association between maternal protein intake with foetal growth parameters

Foetal measurements against protein intake category	N	Mean foetal measurements (mm)	Standard deviation	p value
Average head-circumference for taking				
<50 g/d	28	0.0181	0.02312	
50-70 g/d	77	0.0280	0.07748	0.006
>70 g/d	47	0.0176	0.05661	
Total	152	0.0229	0.06418	
Average abdomen-circumference for taking				
<50 g/d	28	0.0217	0.02615	
50-70 g/d	77	0.0207	0.07726	0.013
>70 g/d	46	0.0207	0.06019	
Total	151	0.0259	0.06525	
Average femur length for taking				
<50 g/d	28	0.0048	0.00576	
50-70 g/d	78	0.0075	0.01963	0.004
>70 g/d	47	0.0045	0.01251	
Total	153	0.0061	0.01583	
Average biparietal diameter for taking				
<50 g/d	27	0.0058	0.00667	
50-70 g/d	78	0.0080	0.02170	0.003
>70 g/d	47	0.0051	0.01547	
Total	152	0.0067	0.01795	

We found significant and better tendency of foetal growth in certain measurements (FL, BPD, AC, and HC) with the second category of milk and protein intake. The relations between milk intake and foetal growth measurements and also birth parameters as seen in our study may be due to supply of micronutrients. It is to be noted that the whole diet assessment and data collection were carried out in the last trimester of pregnancy. Besides, no significant gender difference was seen among maternal milk and protein consumption and foetal growth. Literature shows the relationship between maternal milk and protein intake by mothers with birth parameters. In studies on humans, Chang *et al.* (14) explored the impact of maternal dairy intake on femur length of foetus in pregnant African-American adolescents retrospectively. The study showed that high intake of dairy products by mothers is significantly associated with greater femur length of foetus. The present study also shows the positive association with four foetal traits prospectively.

Since milk contains other nutrients essential for growth of bones, our results may be due to at least nutrients other than calcium. On the other hand, 2 prospective studies (17,18) and a case-control study (19) were unable to detect significant associations between birthweight and small-for-gestational age (SGA) and maternal milk intake. Numbers of retrospective studies detected that high calcium intake in the form of dairy products in early life is positively associated with greater bone mass in adult life (20-23). Studies examining dietary patterns associated with low calcium intake have shown that diets deficient in calcium are also low in other nutrients (24).

SF Olsen (13) remarks that maternal milk intake during pregnancy is associated with an increased mean of birth parameters in the offspring. In addition, they observed that protein from milk is related to birthweight, which suggests that the potentially causative constituents in milk are unlikely to be part of the lipid component of milk. Therefore, there is possibility that IGF-I or other peptide hormones may be the underlying factor accounting for the association between milk and birthweight. However, we did not measure the IGF-I level in our study but we presumed that there is a connecting link between growth and IGF-I via maternal diet during pregnancy.

In a prospective study in India (25), birthweight, birth-length, head-circumference, and placental weight were directly associated with frequency of milk intake assessed at early gestation. Another prospective study on Canadian women (26) also showed a direct effect on birthweight only but no association was seen with birth-length or head-circumference. Moreover, finding from another study showed that intake of excess dietary protein throughout gestation caused lower birthweight, similar to animals fed the low-protein diet (6). The result of rodent studies (4-6) also indicated that increased protein intake during gestation has negative impact on birth parameters, although the findings are inconsistent. Epidemiological studies (1-3) observed that increased protein consumption during gestation leads to foetal growth retardation. High-protein diet increases dietary thermogenesis and allows less availability of energy that may relatively explain the induced lower birthweight (27). The possible reason emerged from experimental studies for growth retardation with low-protein diet might be lack of indispensible amino acid (28) that is associated with reduced nutrient supply (29,30). In addition, aberrant concentration of other metabolites and hormones, such as insulin, leptin, and IGF-I (30,31) might be a related factor for impairment of foetal growth with low-protein diet. It is also reported that increased serum IGF-I in milk enhanced the bone formation (32).

**Table 5.** Regression coefficient and confidence interval for slope

Parameter	Model 1			Model 2		
	B_1_ (SE)	2 B_2_ (SE)	95% CI	B_1_ (SE)	2 B_2_ (SE)	95% CI
HC	2.34 (0.105)	-0.052 (0.004)	(-0.059849, -0.04416)	2.068 (0.087)	-0.042 (0.004)	(-0.03416, -0.04984)
AC	1.507 (0.091)	-0.016 (0.004)	(-0.02384, -0.00816)	1.6 (0.135)	-0.02 (0.004)	(-0.02784, -0.01216)
FL	0.468 (0.032)	-0.008 (0.002)	(-0.01192, -0.00408)	0.352 (0.037)	-0.004 (0.0012)	(-0.006352, -0.001648)
BPD	0.602 (0.035)	-0.012 (0.002)	(0.01592, -0.000808)	0.503 (0.031)	-0.008 (0.002)	(-0.01192, -0.00408)

Our findings are also in agreement with longitudinal studies on adolescents, children of different ages, and infants, in which dairy protein intake is related to growth velocity of height and offspring's birthweight as well (33-38). Thus, part of this relationship is thought to be explained, in part, by another mechanism whereby consumption of dairy promotes growth during gestational period, childhood, and pre-pubertal and pubertal periods. However, our study did not measure the proportion of protein derived from milk or dairy products and planned to exhibit potential role of milk in growth while it is widely known that IGF-I in milk is higher than other fermented dairy products. Long-term consumption of a low-protein, low-calorie diet is associated with low plasma growth factors and hormones. Low-protein diet is associated with a decrease in circulating IGF-I (39).

These findings indicate that a potential factor in the milk and protein has growth-promoting activity during prenatal period and after birth till puberty. It is planned to see the more point-wise role of milk and protein together in quadratic regression model, which more precisely can exhibit the growth differences of foetus between two categories of lower and excess intake of milk-protein. So, regression model best described the pattern of growth and velocity of growth. Growth pattern of foetus in Model 1 (lower milk-protein intake) shows to have more mode of decline compared to Model 2 (excess milk-protein intake), in which better tendency of growth is observed. In addition, there is no significant gender difference as seen in regression model.

There is large evidence, mostly on the rate of foetal growth in different populations through the formulation of foetal centiles, although a very few studies have been published on the growth velocity of foetal biometrical measurements with the effect of maternal dietary intake during gestational period. In a retrospective cohort of adolescent African pregnant women (14), high maternal dairy intake was significantly associated with greater femur length of foetus. A prospective study in India showed birthweight, birth-length, head-circumference, and placental weight were directly associated with frequency of milk intake assessed at 18 weeks of gestation but no associations were observed for milk intake assessed at 28 weeks, with abdominal circumference, dairy protein, and birthweight (25).

### Conclusions

Adequate consumption of milk and protein during gestational period showed enhancement of foetal growth.

In accordance with the result of quadratic regression, foetal growth parameters revealed that three of four foetal anthropometric parameters in Model 2 (high milk-protein intake) exert better tendency of growth in contrast to Model 1 (low milk-protein intake). There is no significant difference in abdomen-circumference of foetus among these two models.

The findings demonstrate that contribution of common nutrients or other nutritional factors present in milk and protein promotes the foetal growth. Therefore, it emphasizes the importance of maternal nutritional intake and availability of nutrients contributing to adequate foetal growth.
